# A prediction model on rockburst intensity grade based on variable weight and matter-element extension

**DOI:** 10.1371/journal.pone.0218525

**Published:** 2019-06-26

**Authors:** Jianhong Chen, Yi Chen, Shan Yang, Xudong Zhong, Xu Han

**Affiliations:** School of Resource and Safety Engineering, Central South University, Changsha, Hunan, China; Northeast Electric Power University, CHINA

## Abstract

Rockburst is a common dynamic disaster in deep underground engineering. To accurately predict rockburst intensity grade, this study proposes a novel rockburst prediction model based on variable weight and matter-element extension theory. In the proposed model, variable weight theory is used to optimize the weights of prediction indexes. Matter-element extension theory and grade variable method are used to calculate the grade variable interval corresponding to the classification standard of rockburst intensity grade. The rockburst intensity grade of Engineering Rock Mass is predicted by rock burst intensity level variable and the interval. Finally, the model is tested by predicting the rockburst intensity grades of worldwide several projects. The prediction results are compared with the actual rockburst intensity grades and the prediction results of other models. The results indicate that, after using variable weight theory and grade variable method, the correct rate of prediction results of matter-element extension model is improved, and the safety of the prediction results is also enhanced. This study provides a new way to predict rock burst in underground engineering.

## Introduction

Rockburst is the common energy release of elastic deformation with sudden, uncontrollable and destructive damage in deep mining and underground space development, which is manifested as the fracture and instantaneous projection of surrounding rock mass [1–3]. Strong rockbursts may cause significant losses to underground engineering, such as the failure of the supporting mechanism and equipment, casualty and construction delay [4,5]. Therefore, it is significant to predict and alarm the rockburst in underground engineering [6,7]. The research on the mechanism of rockburst has been studied from different perspectives with scientific rockburst criteria and prediction method [8]. Xie H P [9] and Cai M F [10,11] obtained the rockburst criteria based on the energy principle by studying the internal relationship between energy accumulation, dissipation, release, rock strength and overall failure in the process of rock deformation and failure. Arteiro A [12] summarized the critical energy value of rockburst through triaxial test and used it as the criteria of rockburst prediction. Jiang Q [13]et al. proposed that local energy release rate (LERR) is a new energy index to simulate rockburst conditions, contributing to understanding the rockburst mechanism from the perspective of energy. He M C [14,15], Kuksenko V S [16] et al. obtained the rockburst prediction criteria by analyzing the characteristics of acoustic emission signal in the rock failure. Feng X T [17], Ma T H [18], Liu X H [19], Chen Z H [20] et al. used microseismic monitoring technology to explore the relationship between the spatiotemporal evolution of microseismic signals and rockburst, and obtained the criteria for rockburst prediction and early warning based on the features of microseismic events. Sharan S K [21] proposed a finite element model for predicting the potential occurrence of rockburst in underground caverns. Mole-Coulomb failure criteria[22] and Hoek–Brown failure criteria[23] was used as rockburst criteria. These studies on rockburst criteria enrich the understanding of rockburst mechanism. However, due to the complex rock mass structure and geological environment in the underground engineering, rock burst prediction by a single rock burst criterion is usually less accurate.

To improve the correct rate of prediction, the information of rockburst reflected by various rockburst criteria have been synthesized by the relevant mathematical theory, and many rockburst prediction and early warning models have been established. For example, Zhou J [24,25] used Fisher discriminant theory to construct a Fisher analysis discriminant model for rockburst classification prediction, which can accurately predict rockburst in deep tunnels and some coal mines. Guo L [26] used the theory of neural network to construct the BP neural network prediction model of rockburst prediction. A large amount of rockburst data were required for the model training, otherwise the correct rate of prediction and the application scope were limited. Liu Z J [27] used the fuzzy probability theory to establish a new fuzzy probability model for predicting the occurrence and intensity of rockburst. The fuzzy weight was introduced in this model, and the limitation of traditional fuzzy probability model was overcame to a certain extent in practical engineering. Gao W [28] constructed a rockburst prediction model by ant colony clustering algorithm. Engineering analogy was performed to realize the automatic classification of rockburst in this model, while prediction accuracy still needed to be further improved. Pei Q T [29] considered the complex relationship between rockburst and its influencing factors as a grey system, optimized the grey whitening weight function, and established an improved grey evaluation model for rockburst prediction. The model greatly overcame the problems of multiple intersections of grey types and abnormality, and improved the prediction accuracy. Based on Projection Pursuit (PP), Particle Swarm Optimization (PSO) and Logistic Curve Function (LCF), a particle swarm projection pursuit model for rockburst prediction was constructed by Xu F [30], ZHOU Xuanchi [31] et al. Particle swarm optimization was used to optimize the parameters of projection index function and LCF, guaranteeing the accuracy of model parameters and prediction accuracy. However, with the increasing number of prediction indexes, the optimization of model parameters becomes more difficult. Zhou J [32–34] used a large number of rockburst data to compare the learning ability of 11 supervised learning algorithms, including linear discriminant analysis (LDA), partial least-squares discriminant analysis (PLSDA), quadratic discriminant analysis (QDA), multilayer perceptron neural network (MLPNN), support vector machine (SVM), random forest (RF), gradient-boosting machine (GBM) and so on. Limitations of these algorithms were also analyzed by Zhou J. In addition, the commonly used models for rockburst prediction include evidence theory model [35], set pair analysis theory model [36], efficacy coefficient method model [37], normal cloud model [38]. Although these models have been applied to the engineering, there are still some defects in the models, such as the difficult fusion of conflicting indicators in evidence chain model; the randomness of forecasting cannot be reflected by the failure of set pair analysis model; the difficult obtaining of satisfactory and unsatisfactory values in efficacy coefficient method; and the gap between forecasting results and actual ones caused by the over ideal distribution of indicators in normal cloud model. Therefore, it is necessary to improve the existing rockburst prediction theories and models.

Compared with the extension prediction model in the previous study, this paper proposes a new rockburst intensity measurement model for underground engineering by variable weight theory and matter-element extension theory. In the proposed model, variable weight theory is introduced to optimize the constant weight of predictors so as to improve the rationality of weighting deamination. Grade variable method and matter-element extension method are combined to establish the grade variable interval of rockburst intensity prediction, corresponding to the classification standard of rockburst intensity grade. The rockburst intensity grade is determined in line with grade variable interval of rockburst intensity. Because the grade variable comprehensively reflects the comprehensive extension correlation information of each rockburst intensity grade, and the correct rate of rockburst intensity prediction can be improved.

## Theoretical framework

### 2.1 The matter-element extension theory

In the matter-element extension theory, the change of objects indicates the exploitation, and the change possibility is called extensibility, and the extensibility of objects is described by the matter-element extension. If an object *S* has the feature *y*, the eigenvalue of feature *y* is expressed by *v*, then the ordered triple *R* = (*S*, *y*, *v*) is used to describe the basic-element of the object, abbreviated as matter-element. If a matter *S* has *n* features, the corresponding eigenvalues of features *y*_1_, *y*_2_, ⋯, *y*_*n*_ are *v*_1_, *v*_2_, ⋯, *v*_*n*_ respectively. The matter-element describing the Subject *S* is denoted as *R* [39].

$$R=(S,y_{i},v_{i})=\left[\begin{array}{ccc} S & y_{1} & v_{1}\\ & y_{2} & v_{2}\\ & \cdots & \cdots \\ & y_{n} & v_{n} \end{array}\right]$$ (1)

The matter-element classical domain of object *S* is *R*_*j*_.

$$R_{j}=(S_{j},y_{i},V_{ji})=\left[\begin{array}{ccc} S_{j} & y_{1} & V_{j1}\\ & y_{2} & V_{j2}\\ & \cdots & \cdots \\ & y_{n} & V_{jn} \end{array}\right]$$ (2)

The matter-element joint domain of object *S* is *R*_0_.

$$R_{0}=(S_{0},y_{i},V_{0i})=\left[\begin{array}{ccc} S_{0} & y_{1} & V_{01}\\ & y_{2} & V_{02}\\ & \cdots & \cdots \\ & y_{n} & V_{0n} \end{array}\right]$$ (3)

Where *S* is the object, *v*_*i*_ is the eigenvalue, *S*_*j*_ is the object corresponding to feature greades of *j*, *j*∈{1,⋯,*m*}, *m* is the number of feature greades, *y*_*i*_ is the features of object *i*, *i*∈{1,⋯,*n*}, *n* is the number of features, *V*_*ji*_ is the eigenvalues range of *S*_*j*_ about *y*_*i*_, *S*_0_ is the object corresponding to overall feature grades, *V*_0*i*_ is the eigenvalues range of *S*_0_ about *y*_*i*_.

According to the extension set and extension distance [40], extending "distance" to "extension distance", the real variable function in classical mathematics is extended to correlation function then the extension distance formula of feature *y*_*i*_ in the object *S* in regard to feature grade *j* can be expressed as:

$$\{\begin{array}{c} \rho (v_{i},V_{ji})=\left|v_{i}-\frac{a_{ji}+b_{ji}}{2}\right|-\frac{b_{ji}-a_{ji}}{2}\\ \rho (v_{i},V_{0i})=\left|v_{i}-\frac{a_{0i}+b_{0i}}{2}\right|-\frac{b_{0i}-a_{0i}}{2} \end{array}$$ (4)

where *v*_*i*_ is the object feature *i*, *V*_*ji*_ is the value range of *S*_*j*_ in regard to feature *y*_*i*_, *V*_*ij*_ = [*a*_*ij*_,*b*_*ij*_], *V*_0*i*_ is the value range of *S*_0_ with respect to feature *y*_*i*_, *V*_0*i*_ = [*a*_0*i*_,*b*_0*i*_], *ρ*(*v*_*i*_,*V*_*ji*_) is the extension distance of feature *y*_*i*_ with respect to the classical domain of *j* feature grade, and *ρ*(*v*_*i*_,*V*_0*i*_) is the extension distance of feature *y*_*i*_ with respect to joint domain.

If *v*_*i*_∊*V*_*0i*_, the elementary function is obtained for calculating the feature correlation [41].

$$S_{j}(v_{i})=\{\begin{array}{ll} \frac{\rho (v_{i},V_{ji})}{\rho (v_{i},V_{0i})‐\rho (v_{i},V_{ji})} & \rho (v_{i},V_{0i})‐\rho (v_{i},V_{ji})\neq 0,v_{i}\in V_{0i}\\ ‐\rho (v_{i},V_{ji})+1 & \rho (v_{i},V_{0i})‐\rho (v_{i},V_{ji})=0,v_{i}\in V_{ij}\\ 0 & \rho (v_{i},V_{0i})‐\rho (v_{i},V_{ji})=0,v_{i}\notin V_{ij},v_{i}\in V_{0i} \end{array}$$ (5)

where *S*_*j*_(*v*_*i*_) is the feature correlation of the feature *y*_*i*_ of the object *S* with respect to the feature grade *j*. Combining with the feature weight vector *W*, the comprehensive correlation degrees of the object *S* with respect to the feature grade *j* is calculated as follows [42].

$$S_{j}(V_{j})=\sum_{i=1}^{n}w_{i}S_{j}(v_{i})$$ (6)

Where *w*_*i*_ is the weight coefficient of the feature *y*_*i*_, *S*_*j*_(*V*_*j*_) is the comprehensive correlation degrees between the object *S* and the feature grade *j*, *S*_*j*_(*v*_*i*_) is the single index correlation of the feature *y*_*i*_ of the object *S* with respect to the feature grade *j*.

### 2.2 Variable weight theory

Since the weight value is always fixed in the process of constant weighting, it can only reflect the relative importance of each feature of objects, ignoring the influence caused by eigenvalue change to the feature weight [43]. In this regard, Zhu Y Z and Li H X improved the variable weight theory [44]. According to the variable weight theory, constructing the variable weight vector can optimize the constant weight of objects, and obtain the feature variable weight.

According to the axiomatic system, the definition of variable weight vectors [45] as follows. There are *m* mappings *W*_*j*_, *j*∈{1,⋯,*m*}, *W*_*j*_: [0,1]^*m*^→[0,1] (*x*_1_, ⋯, *x*_*m*_)→*W*_*j*_ (*x*_1_, ⋯, *x*_*m*_). if *W*_*j*_ satisfies the following three conditions: Condition a. *W*_1_+ ⋯ + *W*_*m*_ = 1. Condition b. *W*_*j*_ for variable *x*_*j*_ continuous. Condition c. *W*_*j*_ is monotone decreasing (or increment) for variable *x*_*j*_. *W*(*X*) *=* {*W*_1_(*X*), ⋯, *W*_*m*_(*X*)} is called the penalty (or excitation) variable weight vector. The definition of feature variable weight vector [45] as follows. There is a mapping *P*:[0,1]^*m*^→[0,1]^*m*^, *X*→*P*(*X*) = {*P*_1_(*X*), ⋯ *P*_*m*_(*X*)}. *P* is called an *m* dimensional feature variable weight vector, if *P* satisfies the following three conditions: Condition 1. *x*_*i*_≥*x*_*j*_→*P*_*i*_(*X*)≤*P*_*j*_(*X*) or *x*_*i*_≥*x*_*j*_→*P*_*i*_(*X*)≥*P*_*j*_(*X*). Condition 2. *P*_*j*_(*X*) is continuous for each variable *x*_*j*_. Condition 3. for any constant weight vector *W*_0_ = {*w*_01_,⋯,*w*_0*m*_}, Eq (7) satisfies the condition a, condition b and condition c, *P* is called a penalty (or excitation) feature variable weight vector.

$$W_{i}(X)=\frac{w_{0i}P_{i}(X)}{\sum_{j=1}^{m}\left(w_{0j}P_{j}(X)\right)},i\in \{1,\cdots ,m\}$$ (7)

Essentially, feature variable weight vector *P*_*j*_(*X*) is a gradient vector of *m*-dimensional real function *B*(*x*) (also known as balanced function) [46], whose calculation equation is as follows.

$$P_{j}(X)=P_{j}(x_{1},\cdots ,x_{m})=\frac{\partial B(x)}{\partial x_{j}}$$ (8)

According to Eqs (7) and (8), the variable weight vector *W*(*X*) of the object *X* can be obtained.

## Prediction model for rockburst intensity grade

### 3.1 Modeling solutions

Due the complex environment around the rock masses in underground engineering, many factors can result in the rockburst. Firstly, based on the analysis of the existing achievement, the scientific predictive index system of rockburst intensity grading was constructed, and the rockburst intensity predictive index grading standard was established. Secondly, the extreme value method was used to standardize the grading standards and the indicators of Engineering Rock Mass. The dimensionality of the forecasting indicators was achieved, and the normalized grading standards for rockburst prediction indicators were obtained. Thirdly, the each grades in the grading standard was regarded as an Ideal Rock Mass; the constant weight of the predictive indexes were calculated by using entropy method to synthesize the index information of the Ideal Rock Masses and the Engineering Rock Masses, and the difference of the predictive indexes was fully considered by using variable weight theory to calculate index variable weight. Finally, based on the matter-element extension theory, the comprehensive correlation degrees between the normalized grading and the Ideal Rock Masses, Engineering Rock Masses, were calculated, and whose grading variables of rockburst were calculated. The prediction grading interval of rockburst intensity was constructed by using the rockburst grading variables of Ideal Rock Masses. The rock burst intensity grade of Engineering Rock Masses can be obtained by judging the grading interval of the variable value of Engineering Rock Masses.

### 3.2 Predictive index system and grading standard

According to a large number of test results and practical experience, the factors causing rockburst can be divided into internal factors and external factors. Internal factors mainly includes compressive strength, shear strength, brittleness, hardness and Poisson's ratio. External factors mainly includes blasting disturbance, tunneling depth, size and shape of underground space. These external factors can damage the integrity and spatial structure of the rock mass, thus changing the stress distribution of the surrounding rock. Rock burst is caused by both internal and external factors. Based on the existing research [47–50], the rockburst intensity predictive index system was established based on the ratio *σ*_*θ*_/*σ*_*c*_ of shear stress to uniaxial compressive strength, the ratio *σ*_*c*_/*σ*_*t*_ of rock uniaxial compressive strength to tensile strength, rock brittleness index *I*_*s*_ and rock elastic energy index *W*_*et*_. The Classification standard of rock burst intensity grading features is shown in Table 1, and the corresponding prediction index grading standard is shown in Table 2.

**Table 1:** Classification standard of rockburst intensity grading features.

Rockburst intensity grades	Feature description	Reference
**I (none)**	No physical phenomena such as tearing, caving and bursting occurs in the surrounding rock wall. The roadway wall remains intact without acoustic emission. Protective measures and monitoring methods are not required.	[50]
**II (slight)**	Loose wall of the surrounding rock with stripping rock and a slight sound of crackling. Protective measures, routine safety monitoring and management are required.	
**III (medium)**	Rock clumps peel off from the chamber or roadway wall with sharp ejection sound frequently, occasional ejection phenomenon. Serious floor heave phenomenon, which is easy to cause personnel injury and mechanical damage. Anti-ejection facilities should be taken into consideration in design and construction, and real-time monitoring should be adopted.	
**IV (severe)**	Large rocks peel off from chamber or roadway wall with sharp ejection sounds continuously, and ejection phenomena. Surrounding rocks deform sharply and a large number of blasting pits appear, which pose a great threat to human and mechanical safety. Corresponding protective measures must be taken to enhance the safety.	

**Table 2:** The corresponding prediction index grading standard.

Grades	*σ*_*θ*_/*σ*_*c*_	*σ*_*c*_/*σ*_*t*_	*I*_*s*_	*W*_*et*_	Reference
**I (none)**	[40.0,53.0]	[0.1,0.3]	[1.5,3.5]	[0,2.0]	[50]
**II (slight)**	(26.7,40.0]	(0.3,0.5]	(3.5,5.5]	(2.0,3.5]	
**III (medium)**	(14.5,26.7]	(0.5,0.7]	(5.5,7.0]	(3.5,5.0]	
**IV (severe)**	[0,14.5)	(0.7,0.9]	(7.0,8.5]	(5.0,6.5]	

To concisely calculate the variable interval corresponding to the rockburst intensity grade, grading criteria of the rockburst intensity is classified into five kinds of Ideal Rock Masses. The predictive indexes of rockburst intensity and their normalized values of these five Ideal Rock Masses are shown in Table 3, and the normalized method of rockburst intensity prediction index is shown in Section 3.3.

**Table 3:** The rockburst intensity predictive index values and corresponding normalized values of Ideal Rock Masses.

Ideal Rock Masses	predictive index values				Normalized value of predictive index			
	*σ*_*θ*_*/σ*_*c*_	*σ*_*c*_/*σ*_*t*_	*I*_*s*_	*W*_*et*_	*σ*_*θ*_*/σ*_*c*_	*σ*_*c*_/*σ*_*t*_	*I*_*s*_	*W*_*et*_
**Rock mass 1**	53.0	0.1	1.5	0	1.00	1.00	1.00	1.00
**Rock mass 2**	40.0	0.3	3.5	2.0	0.76	0.75	0.71	0.69
**Rock mass 3**	26.7	0.5	5.5	3.5	0.50	0.50	0.43	0.46
**Rock mass 4**	14.5	0.7	7.0	5.0	0.27	0.25	0.21	0.23
**Rock mass 5**	0	0.9	8.5	6.5	0.00	0.00	0.00	0.00

## 3.3 Weighting of prediction indicators

Firstly, the Ideal Rock Masses and the Engineering Rock Masses were subjected to dimensionless treatment by the extremum method, so that the dimension of each predictive index was consistent. Then the entropy method was used to determine the constant weight of each predictive index. Finally, the variable weight vector was constructed based on the variable weight theory to optimize the constant weight, and the variable weight of predictive index was obtained. Weighting steps of the rockburst intensity predictive index are as follows:

1. The extreme value method is used to standardize the predictive index *x*_*ij*_ of rock mass *X*_*i*_ and transform it into dimensionless value *v*_*ij*_. The forward predictive index *x*_*ij*_ of rock mass *X*_*i*_ is disposed by Eq (9), and the reverse predictive index *x*_*ij*_ of rock mass *X*_*i*_ is disposed by Eq (10) [51]. *n* is the number of Engineering Rock Masses, *m* is the number of predictive indexes, *i*∈{1, ⋯, *n*}, *j*∈{1, ⋯, *m*}.
  $$v_{ij}=\{\begin{array}{ll} 1 & x_{ij}\geq m_{j}\\ \frac{x_{ij}-m_{j}}{M_{j}-m_{j}} & x_{ij}\in (m_{j},M_{j})\\ 0 & x_{ij}\leq M_{j} \end{array}$$ (9)
  $$v_{ij}=\{\begin{array}{ll} 1 & x_{ij}\leq m_{j}\\ \frac{M_{j}-x_{ij}}{M_{j}-m_{j}} & x_{ij}\in (m_{j},M_{j})\\ 0 & x_{ij}\geq M_{j} \end{array}$$ (10)
  Where *x*_*ij*_ is the predictive index of rock mass *X*_*i*_, *m*_*j*_ is the maximum value of predictive index *x*_*ij*_, *m*_*j*_ represents the minimum value of predictive index *x*_*ij*_, *v*_*ij*_ is the dimensionless value of *x*_*ij*_.
2. The normalized predictive index value *v*_*ij*_ of rock mass *X*_*i*_ is processed and the predictive index information entropy *e*_*j*_ of rock mass *X*_*i*_ is calculated.
  $$r_{ij}=\frac{v_{ij}}{\sum_{i=1}^{n}v_{ij}}$$ (11)
  $$\{\begin{array}{ll} p_{ij}=0 & r_{ij}=0\\ p_{ij}=r_{ij}\ln(r_{ij}) & r_{ij}\neq 0\\ e_{j}=-\frac{1}{\ln(n)}\sum_{i=1}^{n}p_{ij} & \end{array}$$ (12)
  Where *v*_*ij*_ is the normalized predictive index of the rock mass *X*_*i*_, *e*_*j*_ is the information entropy of the predictive index of the rock mass *X*_*i*_, *r*_*ij*_ is the normalized predictive index value of the rock mass *X*_*i*_.
3. the constant weight of predictive index of rock mass *X*_*i*_ is calculated.
  $$w_{j}=\frac{1-e_{j}}{m-\sum_{j=1}^{m}e_{j}}$$ (13)
  Where *w*_*j*_ is the weight of the predictive index of the rock mass *X*_*i*_, *e*_*j*_ is the information entropy of the predictive index of the rock mass *X*_*i*_, and *m* is the number of the predictive indexes of the rock mass *X*_*i*_.
  The constant weight vector *W*_0_ = {*w*_01_,⋯,*w*_0*m*_} can be obtained.
4. According to the variable weight theory, the construction and selection of state variable weight vectors are the core of variable weight implementation. The common feature variable weight vectors include sum type, product type, exponential type and mixed type. Since the exponential feature variable weight vector can determine the appropriate parameters by given different equilibrium forces, it is more flexible [52]. The feature variable weight vectors *P*_*j*_(*X*) = (*P*_*j*_(*x*_1_),⋯,*P*_*j*_(*x*_*m*_)) of exponential type are used. The calculation equation of *P*_*j*_ (*x*_*i*_) is as follows.
  $$P_{j}(x_{i})=\exp(-\beta x_{ij})$$ (14)
  Where *x*_*ij*_ is the predictive index of rock mass *X*_*i*_, *β* indicates the adjustment degree of variable weight vector to the vector equilibrium. *β* is selected according to the actual situation and *β* > 0.

### 3.4 Prediction of the rockburst intensity grade

According to matter-element extension theory and rockburst intensity predictive index standard, the classical domain of rockburst intensity predictive index can be expressed as *R*_*j*_, and the nodal domain as *R*_*0*_. The classical domain *R*_*j*_ expresses the change range of normalized index value in each feature grade of slope rock mass evaluation index, and the nodal *R*_*0*_ expresses the whole range of normalized value of rock mass classification index.

$$R_{j}=\left[\begin{array}{ccccc} & S(4) & S(3) & S(2) & S(1)\\ \sigma_{c}/\sigma_{t} & \left[0.00,0.27\right) & \left[0.27,0.50\right) & \left[0.50,0.76\right) & \left[0.76,1.00\right)\\ \sigma_{\theta }/\sigma_{c} & \left[0.00,0.25\right) & \left[0.25,0.50\right) & \left[0.50,0.75\right) & \left[0.75,1.00\right)\\ I_{s} & \left[0.00,0.21\right) & \left[0.21,0.43\right) & \left[0.43,0.71\right) & \left[0.71,1.00\right)\\ W_{et} & \left[0.00,0.23\right) & \left[0.23,0.46\right) & \left[0.46,0.69\right) & \left[0.69,1.00\right) \end{array}\right]$$ (15)

$$R_{0}=\left[\begin{array}{ccc} S_{0} & \sigma_{c}/\sigma_{t} & \left[0.00,1.00\right]\\ & \sigma_{\theta }/\sigma_{c} & \left[0.00,1.00\right]\\ & I_{s} & \left[0.00,1.00\right]\\ & W_{et} & \left[0.00,1.00\right] \end{array}\right]$$ (16)

According to the grading standard of rockburst intensity, if a predictive index of Engineering Rock Mass is greater than the maximum index, the maximum index is used as the index of Engineering Rock Mass. if it is less than the minimum index, the minimum index is used as the index of Engineering Rock Mass. Then the rockburst intensity predictive indexes of each Engineering Rock Massare normalized according to the Eqs (9) and (10), and normalized predictive index values are obtained. Finally, by Eqs (4), (5) and (6), the comprehensive correlation degrees between each Ideal Rock Mass, each Engineering Rock Mass and the rockburst intensity prediction grading standard are calculated. The grading variable values of rockburst intensity grade of Ideal Rock Masses and Engineering Rock Masses are calculated based on the grading variable method [53]. The equation for calculating grading variables *k* is as follows.

$$P_{j}(V_{j})=\frac{S_{j}(V_{j})-B_{s}}{A_{s}-B_{s}}$$ (17)

$$k=\frac{\sum_{j=1}^{m}jP_{j}(V_{j})}{\sum_{j=1}^{m}P_{j}(V_{j})}$$ (18)

where *S*_*j*_(*V*_*j*_) is the comprehensive correlation degree between *S* and feature grading *j*, *P*_*j*_(*V*_*j*_) is the normalized value of comprehensive correlation, *k* is the value of feature grading of *S*,*B*_*s*_ = min{*S*_*j*_(*V*_*j*_)}, *A*_*s*_ = max{*S*_*j*_(*V*_*j*_)}.

Finally, the grading interval of grading variable is constructed by using the grading variables of Ideal Rock Masses. The rockburst intensity grade of a Engineering Rock Mass is determined by distinguishing the grading interval corresponding to the grading variable of the Engineering Rock Mass.

## Model verification and results analysis

### 4.1 Cases selection

To test the accuracy and safety of the proposed model, several classical rockburst cases of underground engineering at home and abroad [54] are selected for the prediction and analysis.

Case 1: Tianshengqiao II Hydropower Station Headrace Tunnels;
Case 2: Ertan hydropower station 2;
Case 3: Underground Tunnels of Lubuge Hydropower Station;
Case 4: Yuzixi Hydropower Station Headrace Tunnels;
Case 5: Taipingyi Hydropower Station Headrace Tunnels;
Case 6: Pingjin II Hydropower Station Headrace Tunnels;
Case 7: Parallel adit K261+9398 of Erlangshan Tunnels;
Case 8: Zhongnanshan Extra Highway Tunnels of Qinling Mountains;
Case 9: Jiuhuashan Tunnels of Fu’ning Expressway in Fujian Province
Case 10: Heggura Highway Tunnels of NorwayVietas Hydropower Station Headrace Tunnels of Sweden
Case 11: Vietas Hydropower Station Headrace Tunnels of Sweden
Case 12: Japanese Kan-Etsu Tunnels
Case 13: Taipingyi Hydropower Station Headrace Tunnels.

The measured values and normalized values of rockburst prediction in each case are shown in Table 4.

**Table 4:** Predictive index values of rockburst cases in underground engineering at home and abroad.

Project cases	Measured values of predictive indexes				Normalized values of predictive indexes				Reference
	*σ*_*θ*_/*σ*_*c*_	*σ*_*c*_/*σ*_*t*_	*I*_*s*_	*W*_*et*_	*σ*_*θ*_/*σ*_*c*_	*σ*_*c*_/*σ*_*t*_	*I*_*s*_	*W*_*et*_	
**Case 1**	24.0	0.30	5.73	6.6	0.45	0.75	0.40	0.00	[54]
**Case 2**	29.7	0.41	7.26	7.3	0.56	0.61	0.18	0.00	
**Case 3**	27.8	0.23	3.26	7.8	0.53	0.84	0.75	0.00	
**Case 4**	14.8	0.53	7.00	9.0	0.28	0.46	0.21	0.00	
**Case 5**	12.6	0.38	5.30	9.0	0.24	0.65	0.46	0.00	
**Case 6**	18.5	0.82	11.2	3.8	0.35	0.10	0.00	0.42	
**Case 7**	21.2	0.52	6.80	5.5	0.40	0.48	0.24	0.15	
**Case 8**	28.6	0.62	8.40	6.8	0.54	0.35	0.01	0.00	
**Case 9**	24.6	0.52	3.30	7.3	0.46	0.48	0.74	0.00	
**Case 10**	24.1	0.37	9.60	5.0	0.46	0.66	0.00	0.23	
**Case 11**	26.7	0.44	3.60	5.5	0.50	0.58	0.70	0.15	
**Case 12**	22.3	0.39	10.7	5.0	0.42	0.64	0.00	0.23	
**Case 13**	17.3	0.38	7.50	7.6	0.33	0.65	0.14	0.00	

**Fig 1:**
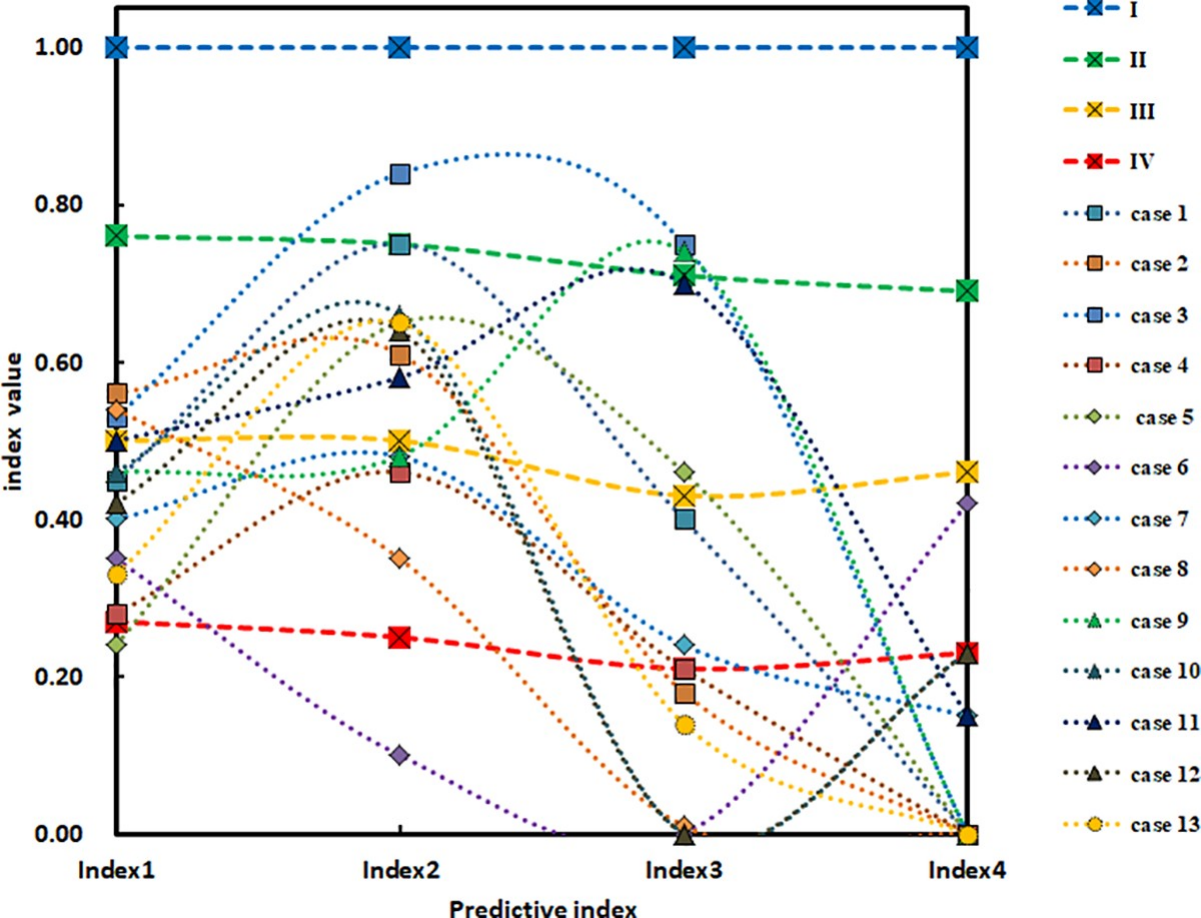
The distribution of predictive indexes for each case. Index 1 is *σ*_*θ*_/*σ*_*c*_, the ratio of rock shear stress and uniaxial compressive strength. Index 2 is *σ*_*c*_/*σ*_*t*_, the ratio of rock uniaxial compressive strength and tensile strength. Index 3 is *I*_*s*_, the brittleness index of rock. Index 4 is *W*_*et*_, the elastic energy index of rock.

Rockburst cases in Table 4 are occurred in different countries and regions, so there is a large difference in the engineering geological conditions. This difference can be reflected in the distribution of the forecasting indicators (Fig 1). At the same time, As shown in Fig 1, there is no regularity between the predictive indexes of different cases in the model verification, and there is a great difference between the predictive indexes of the same case. It is impossible to directly determine the rockburst intensity grade of each case based on a single index. Therefore, these cases can be used to test the correctness of the prediction results of the model.

### 4.2 Calculation and analysis of the weights of predictive indexes

In the exponential variable weight vectors, 𝛽 is the penalty grade, which reflects the adjustment degree of the variable vectors weighting to vector equilibrium of the predictive index. The bigger the value, the more the optimal result is biased toward equilibrium among indexes. 𝛽 = 1 is selected in this paper.

$$P_{j}(X_{i})=\exp(-x_{ij})$$ (19)

**Fig 2:**
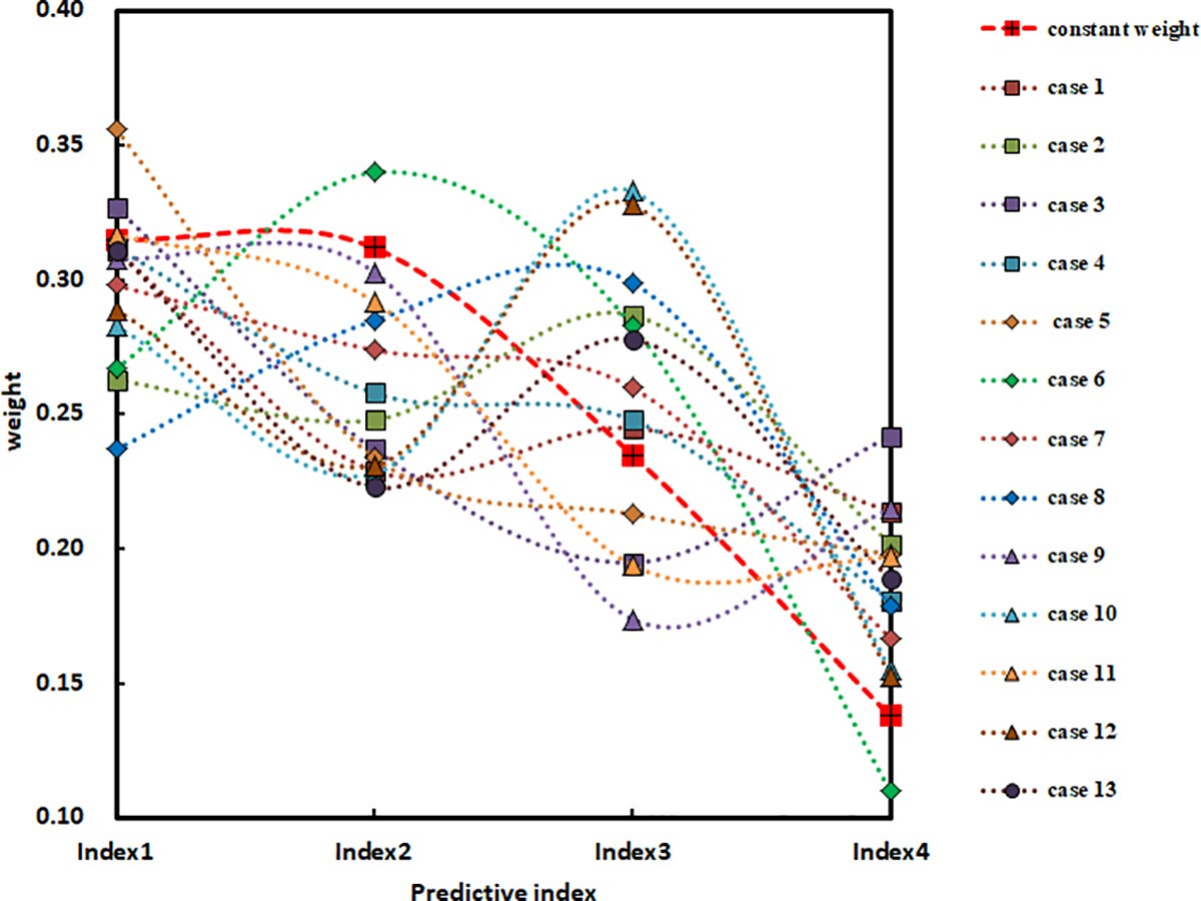
The distribution of variable weights for each case. Index 1 is *σ*_*θ*_/*σ*_*c*_, the ratio of rock shear stress and uniaxial compressive strength. Index 2 is *σ*_*c*_/*σ*_*t*_, the ratio of rock uniaxial compressive strength and tensile strength. Index 3 is *I*_*s*_, the brittleness index of rock. Index 4 is *W*_*et*_, the elastic energy index of rock.

According to the theory of extreme value entropy weighting and variable weight, the constant weight of each rockburst prediction index and the variable weight value of each engineering case are calculated by using Eqs (7), (9)–(13) and (19). The results are shown in Table 5. The constant weight of prediction index synthesizes the prediction indexes in Table 5, which reflects the relative importance of each prediction index. In Fig 2, the relative importance of each prediction index is shown as: that index 1 is more important than Indicator 2, Index 2 is more important than Index 3, and Index 3 is more important than Index 4. Considering the different values of rocks in the prediction index, the variable weight of prediction index is obtained by adjusting the constant weight. It reflects the influence of different values of prediction index on the weight of prediction index. As shown in Fig 2, the relative importance of prediction indexes of different Engineering Rock Masses changes after introducing variable weights. There is no regularity in these changes, indicating the extensive representativeness of the selected rockburst cases in this paper.

**Table 5:** Weight values of predictive indexes for ideal rock mass and engineering cases.

Predictive indexes		*σ*_*θ*_/*σ*_*c*_	*σ*_*c*_/*σ*_*t*_	*I*_*s*_	*W*_*et*_
**Constant weight of predictive indexes**		0.315	0.312	0.235	0.138
**Variable** **Weight of** **Predictive** **indexes**	**Ideal Rock Mass 1**	0.315	0.312	0.235	0.138
	**Ideal Rock Mass 2**	0.309	0.308	0.240	0.144
	**Ideal Rock Mass 3**	0.307	0.306	0.247	0.140
	**Ideal Rock Mass 4**	0.306	0.311	0.242	0.140
	**Ideal Rock Mass 5**	0.315	0.312	0.235	0.138
	**Case 1**	0.311	0.229	0.245	0.214
	**Case 2**	0.263	0.248	0.287	0.202
	**Case 3**	0.327	0.237	0.195	0.242
	**Case 4**	0.312	0.258	0.248	0.181
	**Case 5**	0.356	0.234	0.213	0.198
	**Case 6**	0.267	0.340	0.283	0.110
	**Case 7**	0.298	0.274	0.260	0.167
	**Case 8**	0.237	0.285	0.299	0.179
	**Case 9**	0.308	0.303	0.174	0.215
	**Case 10**	0.283	0.228	0.333	0.155
	**Case 11**	0.317	0.292	0.194	0.197
	**Case 12**	0.289	0.231	0.328	0.153
	**Case 13**	0.311	0.223	0.278	0.189

### 4.3 Prediction results and analysis

According to the Eqs (4)–(6) and (15)-(18), the comprehensive correlation degrees and the rockburst intensity grading variables between each Ideal Rock Mass and each prediction grading standard are calculated as shown in Table 6. The grading variable interval for constructing the rockburst intensity grading is shown in Table 7.As shown in Table 6, prediction index values of rockburst intensity of Ideal Rock Mass come from classification criteria. In this case, the comprehensive correlation degree of a Ideal Rock Mass is a one-to-one correspondence with its rockburst intensity grade. Therefore, according to the comprehensive correlation degree of each ideal rock mass in Table 6, the value of the characteristic grade variable of the calculated rockburst intensity is also a one-to-one correspondence relationship with the rockburst intensity grade, namely, the corresponding relationship in Table 7 is correct.

**Table 6:** The comprehensive correlation degrees and rockburst intensity grading variables of each ideal rock mass.

Ideal Rock Mass	Comprehensive correlation degrees				Grading variables *k*
	*S* (1)	*S* (2)	*S* (3)	*S* (4)	
**Rock mass 1**	1.000	0.000	0.000	0.000	1
**Rock mass 2**	0.000	0.000	-0.487	-0.643	1.662
**Rock mass 3**	-0.347	0.000	0.000	-0.328	2.541
**Rock mass 4**	-0.666	-0.486	0.000	0.000	3.322
**Rock mass 5**	0.000	0.000	0.000	1.000	4

**Table 7:** Rockburst intensity feature grades and grading variable interval.

Feature grade	I	II	III	IV
**Grading variable *k***	[1.00,1.66)	[1.66,2.54)	[2.54,3.32)	[3.32,4]

According to the Eqs (4)—(6), (15)—(18), the comprehensive correlation between the Engineering Rock Mass and the standard grade of rockburst intensity and the variable value of the characteristic grade of rockburst intensity are calculated. Table 8 shows the final prediction results. Model 1 in Table 8 refers to the cloud model based on index distance and uncertainty measurement [50]. Model 2 refers to the normal cloud model [38], Model 3 refers to the set pair theory model [36], and Model 4 refers to the efficiency coefficient method model [37]. Method 1 refers to the matter-element extension model based on variable weight theory with the discrimination criteria of the characteristic grade variables of rockburst intensity. Method 2 refers to the matter-element extension model that uses the variable weight with the discrimination criteria of the comprehensive relevance. Method 3 refers to the matter-element extension model that uses the constant weight with the discrimination criteria of the comprehensive relevance.

**Table 8:** Prediction results 1.

Case	Comprehensive correlation degree				Grading variable *k*	Method 1	Method 2	Method 3	Actual grade
	*S* (1)	*S* (2)	*S* (3)	*S* (4)					
**Case 1**	-0.234	-0.050	-0.053	-0.103	2.893	III	II	II	III
**Case 2**	-0.362	-0.027	-0.136	0.054	3.084	III	IV	II	II
**Case 3**	0.199	-0.091	-0.283	-0.189	1.617	I(close to II)	I	I	I orII
**Case 4**	-0.470	-0.283	0.030	0.093	3.301	III	IV	III	III
**Case 5**	-0.372	-0.080	-0.130	0.063	3.147	III	IV	II	uncertain
**Case 6**	-0.483	-0.365	-0.117	0.576	3.611	IV	IV	IV	III or IV
**Case 7**	-0.542	-0.300	0.100	-0.020	3.199	III	III	III	III
**Case 8**	-0.521	-0.355	-0.183	0.332	3.506	IV	IV	IV	IV
**Case 9**	-0.208	-0.057	-0.050	-0.089	2.925	III	III	II	III or IV
**Case 10**	-0.263	-0.025	-0.040	0.126	3.178	III	IV	II	III
**Case 11**	-0.353	-0.059	-0.202	-0.150	2.860	III(close to II)	II	II	II
**Case 12**	-0.285	-0.020	0.008	0.134	3.158	III(close to IV)	IV	II	III or IV
**Case 13**	-0.448	-0.205	-0.099	0.301	3.377	IV(close to III)	IV	IV	III

In Table 8, prediction results show that the prediction grades of Method 1 on Projects 2, 11 and 13 are lower than the actual grade, and the misjudgment rate is about 23%. In other projects, the prediction results are completely consistent with the actual situation. Method 2 has a lower prediction grade than the actual grade on Projects 4, 10 and 13, a lower prediction grade than the actual grade on Project 2; and a risk misjudgment (higher prediction grade than the actual grade) on Project 1, with a misjudgment rate of about 38%. Method 3 has a lower prediction grade than the actual grade on Project 13; and there are dangerous misjudgments on Projects 1, 9, 10 and 12, the false positive rate is about 38%. Thus, after applying variable weight theory and grading variable method in matter-element extension model, the misjudgment rate of the model is reduced by about 15%, and the occurrence of dangerous misjudgment is reduced.

In Table 9, predicted results show that Model 1 has dangerous misjudgments in Projects 1 and 8. All predictive grades of Model 2 are consistent with the actual grade. The prediction grade of model 3 on Projects 10 and 13 is one grade lower than the actual grade. The predictive grade of Model 4 in Project 2 is lower than the actual grade. Therefore, Method 1 is more secure than Model 1 in predicting results. Compared with Models 2, 3 and 4, the correct rate of Method 1 needs to be improved. However, Method 1 has a relatively high correct rate of prediction results, and the predicted grade is not greater than the actual grade. Hence, Method 1 is of great significance for guiding the safe construction of underground engineering.

**Table 9:** Prediction results 2.

Case	prediction result					Actual grade
	Method 1	Model 1	Model 2	Model 3	Model 4	
**Case 1**	III	II	III	III	III	III
**Case 2**	III	II	II	II	III	II
**Case 3**	I(close to II)	I	I	II	II	I or II
**Case 4**	III	III	III	III or IV	III	III
**Case 5**	III	II	II	uncertain	II	uncertain
**Case 6**	IV	III	III	IV	IV	III or IV
**Case 7**	III	III	III	III	III	III
**Case 8**	IV	III	IV	IV	IV	IV
**Case 9**	III	III	III	III or IV	III	III or IV
**Case 10**	III	III	III	IV	III	III
**Case 11**	(close to II)	II	II	II	II	II
**Case 12**	III(close to IV)	III	III	IV	III	III or IV
**Case 13**	IV (close to III)	III	III	IV	III	III

## Conclusions

1. Taking the ratio of tangential stress to uniaxial compressive strength of rock, uniaxial compressive strength to tensile strength of rock, brittleness index and elastic energy index of rock as prediction index system, the dimension of prediction index is unified by extreme value method. The objective constant weight of prediction index is calculated by entropy weight method, which reflects the relative importance of prediction index. On this basis, the variable weight theory is introduced to fully consider the influence of the difference of rockburst prediction indexes on index weighting, and the variable weight function of penalty characteristic variable weight function is selected to calculate the variable weight of prediction index, so as to improve the rationality of the weighting of prediction index.
2. Based on the grading standards of rockburst intensity index, five ideal rock masses are constructed. The matter-element extension theory and the grade variable method are used to calculate the characteristic grade variables of rockburst intensity for each ideal rock mass. Then value range of the rockburst intensity is constructed, corresponding to the grading standard. Based on this interval, the rockburst grade can be predicted by the variables of characteristic grade of rockburst intensity. This method overcomes the deficiency of matter-element extension model in predicting the rockburst grade with the maximum comprehensive correlation degree.
3. This model is also used to predict the rockburst intensity of several typical underground engineering cases at home and abroad. Results show that in a few projects, the prediction grade of this model is lower than the actual grade, accounting for about 23%. In most projects, the prediction grade is consistent with the actual grade, accounting for about 77%, and there is no large risk misjudgment or the difference between the prediction grade and the actual grade. Compared with the traditional matter-element extension model, the method of distinguishing rockburst intensity grade by the characteristic grade variable of rockburst intensity is proposed in this paper. The correct rate of prediction results is improved by about 15%, and the safety of prediction results is also higher.
4. Compared with normal cloud model, set pair theory model and efficacy coefficient method, the correct rate of the prediction results of this model needs to be further improved. Meanwhile, although the engineering cases collected in this paper are representative, the number is limited, and the reliability of the model needs to be further tested in engineering practice. In addition, in the process of calculating the variable weight of prediction index, the selection of variable weight function is significantly important to the weight calculation. A more reasonable variable weight is required to construct the function according to the characteristics of rockburst index.

## Supporting information

S1 File (DOCX)
