# Supplementary material for: A prediction model on rockburst intensity grade based on variable weight and matter-element extension
Source: PLoS One. 2019 Jun 26;14(6):e0218525. doi: 10.1371/journal.pone.0218525 (PMC6594615; doi:10.1371/journal.pone.0218525)
Supplement: S1 File — (DOCX) [file pone.0218525.s001.docx]

**Table 1. Classification standard of rockburst intensity grading features**

| **Rockburst intensity grades** | **Feature description** | **Reference** |
| --- | --- | --- |
| **I** (none) | No physical phenomena such as tearing, caving and bursting occurs in the surrounding rock wall. The roadway wall remains intact without acoustic emission. Protective measures and monitoring methods are not required. | [50] |
| **II** (slight) | Loose wall of the surrounding rock with stripping rock and a slight sound of crackling. Protective measures, routine safety monitoring and management are required. |  |
| **III** (medium) | Rock clumps peel off from the chamber or roadway wall with sharp ejection sound frequently, occasional ejection phenomenon. Serious floor heave phenomenon, which is easy to cause personnel injury and mechanical damage. Anti-ejection facilities should be taken into consideration in design and construction, and real-time monitoring should be adopted. |  |
| **IV** (severe) | Large rocks peel off from chamber or roadway wall with sharp ejection sounds continuously, and ejection phenomena. Surrounding rocks deform sharply and a large number of blasting pits appear, which pose a great threat to human and mechanical safety. Corresponding protective measures must be taken to enhance the safety. |  |

**Table 2.** **The corresponding prediction index grading standard**

| **Grades** | ***σ_θ_*/*σ_c_*** | ***σ_c_*/*σ_t_*** | ***I_s_*** | ***W_et_*** | **Reference** |
| --- | --- | --- | --- | --- | --- |
| **I** (none ) | [40.0,53.0] | [0.1,0.3] | [1.5,3.5] | [0,2.0] | [50] |

**Table 3.** **The rockburst intensity predictive index values and corresponding normalized values of Ideal Rock Masses**

| **Ideal Rock Masses** | **predictive index values** | | | | **Normalized value of predictive index** | | | |
| --- | --- | --- | --- | --- | --- | --- | --- | --- |
|  | ***σ_θ_/σ_c_*** | ***σ_c_*/*σ_t_*** | *I_s_* | *W_et_* | ***σ_θ_/σ_c_*** | ***σ_c_*/*σ_t_*** | *I_s_* | *W_et_* |
| **Rock mass 1** | 53.0 | 0.1 | 1.5 | 0 | 1.00 | 1.00 | 1.00 | 1.00 |
| **Rock mass 2** | 40.0 | 0.3 | 3.5 | 2.0 | 0.76 | 0.75 | 0.71 | 0.69 |
| **Rock mass 3** | 26.7 | 0.5 | 5.5 | 3.5 | 0.50 | 0.50 | 0.43 | 0.46 |
| **Rock mass 4** | 14.5 | 0.7 | 7.0 | 5.0 | 0.27 | 0.25 | 0.21 | 0.23 |
| **Rock mass 5** | 0 | 0.9 | 8.5 | 6.5 | 0.00 | 0.00 | 0.00 | 0.00 |

Case 1: Tianshengqiao II Hydropower Station Headrace Tunnels;

Case 2: Ertan hydropower station 2;

Case 3: Underground Tunnels of Lubuge Hydropower Station;

Case 4: Yuzixi Hydropower Station Headrace Tunnels;

Case 5: Taipingyi Hydropower Station Headrace Tunnels;

Case 6: Pingjin II Hydropower Station Headrace Tunnels;

Case 7: Parallel adit K261+9398 of Erlangshan Tunnels;

Case 8: Zhongnanshan Extra Highway Tunnels of Qinling Mountains;

Case 9: Jiuhuashan Tunnels of Fu’ning Expressway in Fujian Province

Case 10: Heggura Highway Tunnels of NorwayVietas Hydropower Station Headrace Tunnels of Sweden

Case 11: Vietas Hydropower Station Headrace Tunnels of Sweden

Case 12: Japanese Kan-Etsu Tunnels

Case 13: Taipingyi Hydropower Station Headrace Tunnels.

**Table 4. predictive index values of rockburst cases in underground engineering at home and abroad**

| **Project cases** | **Measured values of predictive indexes** | | | | **Normalized values of predictive indexes** | | | | **Reference** |
| --- | --- | --- | --- | --- | --- | --- | --- | --- | --- |
|  | ***σ_θ_*/*σ_c_*** | ***σ_c_*/*σ_t_*** | ***I_s_*** | ***W_et_*** | ***σ_θ_*/*σ_c_*** | ***σ_c_*/*σ_t_*** | ***I_s_*** | ***W_et_*** |  |
| **Case 1** | 24.0 | 0.30 | 5.73 | 6.6 | 0.45 | 0.75 | 0.40 | 0.00 | [54] |
| **Case 2** | 29.7 | 0.41 | 7.26 | 7.3 | 0.56 | 0.61 | 0.18 | 0.00 |  |
| **Case 3** | 27.8 | 0.23 | 3.26 | 7.8 | 0.53 | 0.84 | 0.75 | 0.00 |  |
| **Case 4** | 14.8 | 0.53 | 7.00 | 9.0 | 0.28 | 0.46 | 0.21 | 0.00 |  |
| **Case 5** | 12.6 | 0.38 | 5.30 | 9.0 | 0.24 | 0.65 | 0.46 | 0.00 |  |
| **Case 6** | 18.5 | 0.82 | 11.2 | 3.8 | 0.35 | 0.10 | 0.00 | 0.42 |  |
| **Case 7** | 21.2 | 0.52 | 6.80 | 5.5 | 0.40 | 0.48 | 0.24 | 0.15 |  |
| **Case 8** | 28.6 | 0.62 | 8.40 | 6.8 | 0.54 | 0.35 | 0.01 | 0.00 |  |
| **Case 9** | 24.6 | 0.52 | 3.30 | 7.3 | 0.46 | 0.48 | 0.74 | 0.00 |  |
| **Case 10** | 24.1 | 0.37 | 9.60 | 5.0 | 0.46 | 0.66 | 0.00 | 0.23 |  |
| **Case 11** | 26.7 | 0.44 | 3.60 | 5.5 | 0.50 | 0.58 | 0.70 | 0.15 |  |
| **Case 12** | 22.3 | 0.39 | 10.7 | 5.0 | 0.42 | 0.64 | 0.00 | 0.23 |  |
| **Case 13** | 17.3 | 0.38 | 7.50 | 7.6 | 0.33 | 0.65 | 0.14 | 0.00 |  |

**Table 5. Weight values of predictive indexes for Ideal Rock Mass and Engineering Cases**

| **Predictive indexes** | | ***σ_θ_*/*σ_c_*** | ***σ_c_*/*σ_t_*** | ***I_s_*** | ***W_et_*** |
| --- | --- | --- | --- | --- | --- |
| **Constant weight of predictive indexes** | | 0.315 | 0.312 | 0.235 | 0.138 |
| **Variable**  **Weight of**  **Predictive**  **indexes** | **Ideal Rock Mass 1** | 0.315 | 0.312 | 0.235 | 0.138 |
|  | **Ideal Rock Mass 2** | 0.309 | 0.308 | 0.240 | 0.144 |
|  | **Ideal Rock Mass 3** | 0.307 | 0.306 | 0.247 | 0.140 |
|  | **Ideal Rock Mass 4** | 0.306 | 0.311 | 0.242 | 0.140 |
|  | **Ideal Rock Mass 5** | 0.315 | 0.312 | 0.235 | 0.138 |
|  | **Case 1** | 0.311 | 0.229 | 0.245 | 0.214 |
|  | **Case 2** | 0.263 | 0.248 | 0.287 | 0.202 |
|  | **Case 3** | 0.327 | 0.237 | 0.195 | 0.242 |
|  | **Case 4** | 0.312 | 0.258 | 0.248 | 0.181 |
|  | **Case 5** | 0.356 | 0.234 | 0.213 | 0.198 |
|  | **Case 6** | 0.267 | 0.340 | 0.283 | 0.110 |
|  | **Case 7** | 0.298 | 0.274 | 0.260 | 0.167 |
|  | **Case 8** | 0.237 | 0.285 | 0.299 | 0.179 |
|  | **Case 9** | 0.308 | 0.303 | 0.174 | 0.215 |
|  | **Case 10** | 0.283 | 0.228 | 0.333 | 0.155 |
|  | **Case 11** | 0.317 | 0.292 | 0.194 | 0.197 |
|  | **Case 12** | 0.289 | 0.231 | 0.328 | 0.153 |
|  | **Case 13** | 0.311 | 0.223 | 0.278 | 0.189 |

**Table 6. The comprehensive correlation degrees and rockburst intensity grading variables of each Ideal Rock Mass**

| **Ideal Rock Mass** | **Comprehensive correlation degrees** | | | | Grading variables *k* |
| --- | --- | --- | --- | --- | --- |
|  | ***S* (1)** | ***S* (2)** | ***S* (3)** | ***S* (4)** |  |
| **Rock mass 1** | 1.000 | 0.000 | 0.000 | 0.000 | 1 |
| **Rock mass 2** | 0.000 | 0.000 | -0.487 | -0.643 | 1.662 |
| **Rock mass 3** | -0.347 | 0.000 | 0.000 | -0.328 | 2.541 |
| **Rock mass 4** | -0.666 | -0.486 | 0.000 | 0.000 | 3.322 |
| **Rock mass 5** | 0.000 | 0.000 | 0.000 | 1.000 | 4 |

**Table 7. Rockburst intensity feature grades and grading variable interval**

| **Feature grade** | **I** | **II** | **III** | **IV** |
| --- | --- | --- | --- | --- |
| **Grading variable *k*** | [1.00,1.66) | [1.66,2.54) | [2.54,3.32) | [3.32,4] |

**Table 8. Prediction results 1**

| **Case** | **Comprehensive correlation degree** | | | | **Grading variable *k*** | **Method 1** | **Method 2** | **Method 3** | **Actual grade** |
| --- | --- | --- | --- | --- | --- | --- | --- | --- | --- |
|  | ***S* (1)** | ***S* (2)** | ***S* (3)** | ***S* (4)** |  |  |  |  |  |
| **Case 1** | -0.234 | -0.050 | -0.053 | -0.103 | 2.893 | III | II | II | III |
| **Case 2** | -0.362 | -0.027 | -0.136 | 0.054 | 3.084 | III | IV | II | II |
| **Case 3** | 0.199 | -0.091 | -0.283 | -0.189 | 1.617 | I(close to II) | I | I | I orII |
| **Case 4** | -0.470 | -0.283 | 0.030 | 0.093 | 3.301 | III | IV | III | III |
| **Case 5** | -0.372 | -0.080 | -0.130 | 0.063 | 3.147 | III | IV | II | uncertain |
| **Case 6** | -0.483 | -0.365 | -0.117 | 0.576 | 3.611 | IV | IV | IV | III or IV |
| **Case 7** | -0.542 | -0.300 | 0.100 | -0.020 | 3.199 | III | III | III | III |
| **Case 8** | -0.521 | -0.355 | -0.183 | 0.332 | 3.506 | IV | IV | IV | IV |
| **Case 9** | -0.208 | -0.057 | -0.050 | -0.089 | 2.925 | III | III | II | III or IV |
| **Case 10** | -0.263 | -0.025 | -0.040 | 0.126 | 3.178 | III | IV | II | III |
| **Case 11** | -0.353 | -0.059 | -0.202 | -0.150 | 2.860 | III(close to II) | II | II | II |
| **Case 12** | -0.285 | -0.020 | 0.008 | 0.134 | 3.158 | III(close to IV) | IV | II | III or IV |
| **Case 13** | -0.448 | -0.205 | -0.099 | 0.301 | 3.377 | IV(close to III) | IV | IV | III |

**Table 9. Prediction results 2**

| **Case** | **prediction result** | | | | | **Actual grade** |
| --- | --- | --- | --- | --- | --- | --- |
|  | **Method 1** | **Model 1** | **Model 2** | **Model 3** | **Model 4** |  |
| **Case 1** | III | II | III | III | III | III |
| **Case 2** | III | II | II | II | III | II |
| **Case 3** | I(close to II) | I | I | II | II | I or II |
| **Case 4** | III | III | III | III or IV | III | III |
| **Case 5** | III | II | II | uncertain | II | uncertain |
| **Case 6** | IV | III | III | IV | IV | III or IV |
| **Case 7** | III | III | III | III | III | III |
| **Case 8** | IV | III | IV | IV | IV | IV |
| **Case 9** | III | III | III | III or IV | III | III or IV |
| **Case 10** | III | III | III | IV | III | III |
| **Case 11** | (close to II) | II | II | II | II | II |
| **Case 12** | III(close to IV) | III | III | IV | III | III or IV |
| **Case 13** | IV (close to III) | III | III | IV | III | III |
